# DOG1-Positive Primary Mesenteric Leiomyosarcoma: Report of a Case and Review of the Literature

**DOI:** 10.7759/cureus.25263

**Published:** 2022-05-23

**Authors:** Nektarios Koufopoulos, Vasileia Damaskou, Vasiliki Siozopoulou, Panagiotis Kokoropoulos, Alina-Roxani Gouloumis, Nikolaos Arkadopoulos, Ioannis G Panayiotides

**Affiliations:** 1 Pathology, Attikon University Hospital, National and Kapodistrian University of Athens, Athens, GRC; 2 Pathology, Antwerp University Hospital, Edegem, BEL; 3 Surgery, Attikon University Hospital, National and Kapodistrian University of Athens, Athens, GRC; 4 Pathology and Laboratory Medicine, Attikon University Hospital, National and Kapodistrian University of Athens, Athens, GRC

**Keywords:** dog1, immunohistochemistry, mesentery, leiomyosarcoma, gastrointestinal stromal tumor

## Abstract

The mesentery constitutes a common location for the metastatic spread of malignant gastrointestinal tumors. Primary mesenteric tumors, on the other hand, are very rare; lymphomas are the most common, followed by benign and malignant mesenchymal tumors. We present a case of a 43-year-old patient operated on for a primary mesenteric leiomyosarcoma with a positive immunostain for DOG1, despite having no *KIT* or *PDGFRa* mutations on molecular analysis. Moreover, we review the pertinent literature.

## Introduction

Leiomyosarcoma (LMS) is a malignant mesenchymal tumor originating from smooth muscle cells or from their precursor mesenchymal stem cells [1]. It usually arises in soft tissue, gastrointestinal tract, retroperitoneum, medium or large veins, and uterus [2,3]. Mesenteric LMS is a rare entity with a reported incidence of 1:350,000 [4], with only 20 immunohistochemically confirmed cases being reported. It is believed to arise from smooth muscle cells of the mesenteric blood vessel wall [2]. It is more often located in the small intestinal mesentery, followed by the transverse and sigmoid mesocolon or the gastrohepatic ligament [2]. The differential diagnosis primarily includes a mesenteric gastrointestinal stromal tumor (GIST). Since treatment and prognosis differ, CD117/KIT and DOG1 immunopositivity are the gold standards for a GIST diagnosis. Nevertheless, we report a DOG1 immunopositivity mesenteric LMS case, the first-ever reported to our best knowledge.

This article was presented as a meeting abstract at the 33rd European Congress of Pathology (ECP 2021) on August 29-31, 2021.

## Case presentation

**Figure 1:**
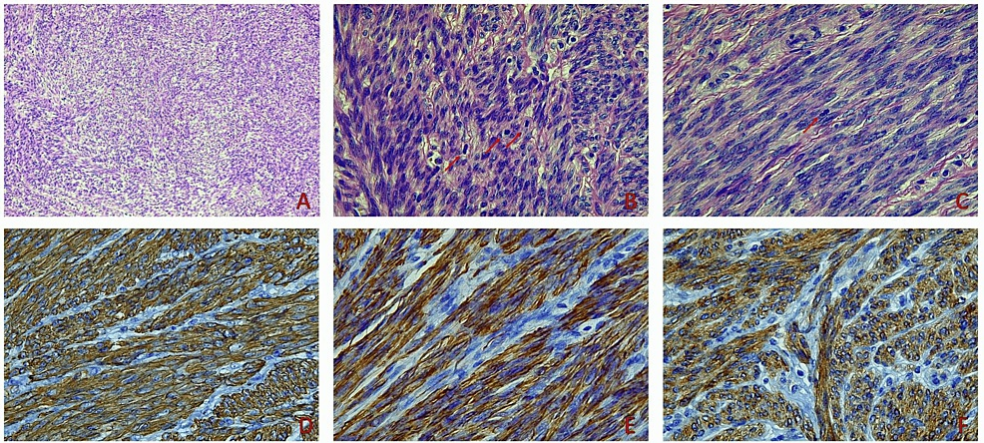
The tumor consisted of atypical spindle cells arranged in fascicles with moderate pleomorphism 10x H&E (A). Up to eight mitoses per 10 high power fields (red arrows) were found, 40x H&E (B,C). Immunohistochemical study showed uniform positivity for SMA, 40x SMA (D), desmin, 40x desmin (E), and DOG1, 40x DOG1 (F) H&E: hematoxylin and eosin stain; SMA: smooth muscle actin

A 43-year-old patient was admitted in March 2020 due to a sizeable mesenteric mass discovered during a previous myomectomy for uterine leiomyomas (performed elsewhere) in October 2019. History was significant for cervical conization due to high-grade squamous intraepithelial lesion (HGSIL) fibroids. On laboratory investigation, inflammatory response markers and cancer tumor markers were within normal limits. The patient underwent a magnetic resonance imaging that revealed a mass with dimensions 4.7x4x3.3 cm that probably originated from the bowel wall or its mesentery with intermediate signal intensity in sequences T1 and T2 and intense inhomogeneous contrast enhancement. The patient was discussed in our local multidisciplinary tumor board (MDT), and the decision was made to proceed with surgical excision of the lesion. We performed an open excision of a mass that arose from the mesentery of the ileum. The tumor was excised with wide margins; it was of whitish color and firm texture. On microscopic examination, the tumor was seen to consist of fascicles of moderately pleomorphic, atypical spindle cells (Figure 1A) with up to eight mitoses per 10 high power fields (Figure 1B, 1C). Immunohistochemically, tumor cells stained positive for smooth muscle actin (SMA) (Figure 1D), desmin (Figure 1E), and DOG1 (Figure 1F), whereas they were negative for CD34 and S100 protein. The proliferation index assessed through immunostaining for Ki67 was estimated as 1%.

Molecular analysis (performed at the Department of Pathology, Antwerp University Hospital, Edegem, Belgium) disclosed no mutations of *KIT* or *PDGFRA* genes. Based on the above findings, a diagnosis of malignant mesenchymal tumor consistent with low-grade leiomyosarcoma of the mesentery was rendered. A review of slides from the previous myomectomy specimens confirmed the diagnosis of leiomyomas.

**Figure 2:**
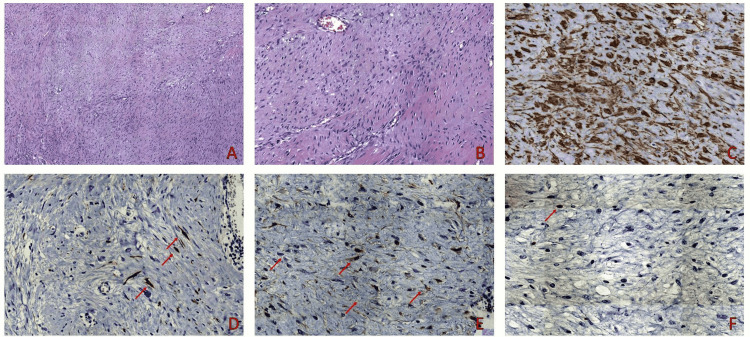
Tumor cells were uniform, elongated, spindled shaped with minimal cytologic atypia without mitotic activity set in a collagenous stroma arranged in long sweeping fascicles 04x H&E (A), 10x H&E (B). Immunohistochemically, tumor cells showed diffuse SMA expression 20x SMA (C) and desmin (red arrows) 20x desmin (D), and diffuse cytoplasmic and focal nuclear β-catenin expression (red arrows) 20x β-catenin (E). Ki67 stained less than 1% of tumor nuclei (red arrow) 20x Ki67 ( F). H&E: hematoxylin and eosin stain; SMA: smooth muscle actin

A mass within the transversus abdominis muscle was found four months later during a postoperative check-up. Microscopic examination of the surgical specimen disclosed long, sweeping fascicles of uniform spindle cells with minimal cytologic atypia and low mitotic activity (one mitosis per 10 high-power field (HPF)) set within a collagenous stroma (Figure 2A, 2B). Tumor cells were diffusely positive for SMA (Figure 2C), as well as focally for muscle-specific actin (MSA) and desmin (Figure 2D); moreover, they showed diffuse cytoplasmic and focal nuclear β-catenin expression (Figure 2E), whereas they did not stain for either CD117 or DOG1. The proliferation index (assessed as the percentage of Ki67-positive cells) was estimated at around 1% (Figure 2F).

A diagnosis of abdominal fibromatosis/desmoid was therefore rendered. The MDT decided not to administer adjuvant therapy. At the two-year follow-up since surgical excision, the patient is free from any recurrence of either tumor or metastatic leiomyosarcoma.

## Discussion

In contrast to metastases from gastrointestinal tumors, primary mesenteric tumors are rare, lymphomas being the most common, followed by usually benign mesenchymal tumors [2]. Mesenteric leiomyosarcoma is very rare, with only 20 cases reported [2-18]: six were male and 14 female. Age ranged from 13 to 86 years (median 54 years). Tumor size ranged from 5.3 to 36 cm (median 13.7 cm). Symptoms occur late in the disease course and are non-specific, usually a palpable mass, abdominal distention, and/or pain. Less commonly, abscess, acute intraperitoneal hemorrhage, or obstructive ileus may occur [2]. One patient presented with posterior reversible encephalopathy syndrome [15]. The liver was the most frequent location of metastatic disease. All cases were treated surgically for the primary and five for synchronous or metachronous metastatic lesions. Adjuvant chemotherapy was administered to eight patients.

In seventeen cases, follow-up was available, ranging from 3 to 59 months (median, 21 months). Eight patients died during the follow-up period, usually due to local recurrence or metastasis to the liver and lung. Two patients were alive with disease, and seven were alive with no evidence of disease. The demographic and clinical data are summarized in Table 1.

**Table 1:** Demographic and clinical data of patients with mesenteric LMS. M: male; F: female; N/M: not mentioned; LR: local recurrence; AWD: alive with disease; DOD: died of disease; ANED: alive with no evidence of disease; PRES: posterior reversible encephalopathy syndrome; LMS: leiomyosarcoma

Case	Authors	Year	Age	Gender	Symptoms	Size (cm)	Tumor location	Metastases	Chemo	Outcome (mo)
1	Miettinen et al. [10]	1999	41	M	N/M	N/M	N/M	Liver	N/M	12 AWD
2	Miettinen et al. [10]	1999	86	M	N/M	N/M	N/M	N/M	N/M	3 DOD
3	Fukanaga et al. [11]	2004	62	F	Mass	14	Sigmoid colon	Liver	Yes	10 DOD
4	Simonovich et al. [7]	2006	82	F	Mass, pain	11	Small intestine	Liver	No	24 Alive
5	Iwasaki et al. [12]	2010	13	M	Mass	10	Small intestine	No	No	3 ANED
6	Koczkowska et al. [8]	2013	62	F	No	7.8	Small intestine	Liver, LR	Yes	24 DOD
7	Koczkowska et al. [8]	2013	46	F	Mass	N/M	Sigmoid colon	Liver, LR	Yes	58 DOD
8	Mizobe et al. [13]	2013	65	M	Mass, pain	20	Ascending colon	Liver, LR	Yes	18 DOD
9	Sidhic et al. [5]	2015	33	M	Pain	15	Small intestine	No	N/M	N/M
10	Hamed et al. [9]	2015	49	F	N/M	N/M	Small intestine	Liver	N/M	59 AWD
11	Hamed et al. [9]	2015	46	M	N/M	N/M	Rectum	Liver, lung	N/M	26 DOD
12	Dasgupta et al. [2]	2016	62	F	Fullness	22	Small intestine	No	No	6 ANED
13	Kato et al. [3]	2016	76	F	Mass	14	Descending colon	Liver	No	40 ANED
14	Varghese et al. [6]	2016	40	F	Mass, Pain	11	Descending colon	Liver, lung	Yes	39 ANED
15	Ilias et al. [4]	2017	45	F	Mass	20	Sigmoid colon	No	No	9 ANED
16	Dalal et al. [1]	2017	50	F	Mass	15	Small intestine	No	No	6 ANED
17	Schoucair et al. [14]	2018	52	F	Pain, PRESS	10	Sigmoid colon	Liver, lung, LR	Yes	20 DOD
18	Yoon et al. [15]	2019	62	F	Mass, Pain	25	Descending colon	Omentum, peritoneum	Yes	N/M
19	Affas et al. [16]	2020	68	F	Pain	5.7	Ascending colon	LR	N/M	12 DOD
20	Deshmukh et al. [17]	2021	31	F	Mass	36	Descending colon	No	Yes	15 ANED
21	Present case	2021	43	F	No	5.3	ileum	No	No	24 ANED

The management of LMS of the mesentery depends largely on preoperative imaging [2]. On CT, most solid lesions are neoplastic compared to cystic ones that are typically benign [19]. However, Dalal et al. have reported a case of entirely cystic LMS [2]. Radiologically, a helpful clue to differentiate GIST from sarcomas is the former's less invasive appearance [20]. Percutaneous biopsy tends to be avoided since it may cause skin metastasis and peritoneal dissemination [6]. Thus, precise preoperative diagnosis based just on clinical and radiological findings is difficult [2]. The final and accurate diagnosis can be made only by the pathological examination of the surgical specimen.

In our case, we used an immunohistochemical panel consisting of SMA, desmin, CD117, and DOG1 to confirm the diagnosis. SMA and desmin staining are positive in almost all smooth muscle tumors compared to 31% and 4% of GISTs [21]. Both CD117 and DOG1 are considered relatively sensitive and specific markers for GIST diagnosis [22,23]. Focal DOG1 staining has been reported in almost 50% of the gastrointestinal intramural leiomyomas and imputed to colonization by Cajal cells, whereas smooth muscle cells are not stained. Uterine leiomyomas in the retroperitoneum and peritoneal leiomyomatosis were also focally DOG1 positive to a lesser degree (5/42 and 4/17 cases, respectively) [24]. Focal or diffuse DOG1 positivity has been previously reported in 27% of uterine LMS [25].

However, DOG1 expression has not been previously reported in a mesenteric LMS. In our case, uniform positive staining for SMA, desmin, and DOG1 was confusing. Despite the lack of CD117 immunopositivity, a GIST diagnosis could not be ruled out since DOG1 is a more sensitive marker than CD117 [26]. We, therefore, proceeded with molecular analysis, which showed no *KIT* or *PDGFRa* mutations, thus favoring the diagnosis of mesenteric LMS since only a small fraction (10-15%) of GISTs are devoid of these mutations [27].

Regarding prognosis, LMS of the mesentery has an aggressive clinical course with an unfavorable prognosis [2]. The overall five-year survival rate for abdominal LMS is only between 20% and 30% [19]. Earlier detection by ultrasonography and CT and complete surgical resection with tumor-free margins may improve long-term prognosis [6]. However, early detection is unusual due to the late appearance of symptoms. In several cases, LMS is detected after distant metastases occur [28].

Tumor recurrence can occur within five years in approximately 50% of patients despite adequate local control [29]. Careful postoperative follow-up is needed to detect a recurrence or metastasis for at least five years, with particular attention to the digestive tract, liver, and lung [6,7].

There is no clear precedent for treating mesenteric LMS, probably due to the small number of cases reported [6]. Treatment options depend upon the stage of presentation. For localized tumors, the treatment of choice is surgical excision with a wide margin of healthy tissue to achieve negative margins. Radiotherapy improves local control and preserves function, reduces local recurrence, but does not improve survival [1]. Metastatic disease is considered incurable since adjuvant chemotherapy or radiotherapy are ineffective [7,8].

## Conclusions

Mesenteric LMS is an infrequent entity. Its diagnosis requires a combination of appropriate morphology and immunohistochemistry. As evidenced by the case we present, molecular analysis is necessary to make the correct diagnosis when morphological and immunohistochemical findings are inconclusive.
